# Rush Medical College—Alumni Meeting

**Published:** 1869-03-01

**Authors:** W. C. Hunt

**Affiliations:** Secretary


### PROCEEDINGS OF SOCIETIES.

### *Rush Medical College—Alumni Meeting.*

The first annual meeting of the "Alumni Association of Rush Medical College" was held in the lower lecture room of the College on Feb. 3, 1869, "Commencement day," the President, Prof. E. Powell, in the chair.

Members present, Professor J. V. Z. Blaney, Jos. W. Freer, E. Ingals, M. Gunn and J. P. Ross. Drs. G. C. Paoli, G. J. Avery, N. T. Quales, C. T. Fenn, J. T. Weeks, T. D. Fitch, F. Henrotin, Jr., B. Durham, Theo. W. Stull, Geo. Egbert, D. V. Bachelle, F. A. Emmons, Ben. C. Miller, C. T. Parkes, D. L. Davidson, J. McClure, J. A. M. Gibbs, F. W. Coffin, H. Brownlee, E. S. Lathrop and the graduating class of 1868 and 1869.

The Constitution of the Association was read. The minutes of the meeting for the organization of the Association, Oct. 1, 1867, were read and approved.

On motion of Dr. C. T. Fenn, the Association proceeded to the election of officers for the ensuing year, with the following result:

For President, Dr. Alfred E. Ames, of Minneapolis, Minn.; for Vice President, Dr. G. C. Paoli, of Chicago; for second Vice President, Dr. B. F. Swafford, of Indiana; for Secretary, Dr. W. C. Hunt, of Chicago; for Treasurer, Dr. F. A. Emmons, of Chicago, and for Executive Committee, Drs. S. J. Avery, W. T. Quales and C. T. Parkes, of Chicago.

Dr. J. C. Paoli was then conducted to the chair, and in a few pertinent remarks assumed its duties, when Prof. Blaney was introduced, and as President of the College on behalf of the Faculty, welcomed the Association to the halls of the College, congratulated them upon the auspicious event that, notwithstanding the inclemency of the weather, so many were assembled to do honor to their *Alma Mater*; alluded to the high character and standing of the graduates of Rush Medical College, not only during the late war of the rebellion, but throughout the Northwest and wheresoever dispersed, claiming for them a position in the profession the first among their equals, second to none in the world, and closed with an eloquent tribute to the memory of the late lamented President, Prof. Brainard, reminding the Association that to his genius and untiring efforts is mainly due the proud position the College and the her alumni now occupy, and that he felt assured that all who knew Brainard would keep for him a place in their memories as one who was entitled to and held a position among the first and most eminent surgeons of America, as the most original genius of his age in our country, and as the man who has contributed as much to the advance of surgery as any other member of the profession in America.

Prof. Powell, the retiring President, then delivered the first annual address, which was listened to with deep interest, and at its conclusion, Dr. Avery moved that the thanks of the Association be tendered to Prof. Powell for his very interesting and able address, which was heartily and unanimously carried.

On motion of Dr. C. T. Fenn, the Executive Committee was instructed to provide for an address or report for the next meeting other than the regular annual one of the President.

On motion, Prof. J. W. Freer and Dr. C. T. Fenn were added to the Committee to carry out the object of the last motion.

On motion of Prof. Ingals, the Secretary was instructed to request of the graduates of Rush Medical College wheresoever they may be (through the different medical journals)

to forward to him any history, or writing, or notice, either obituary or otherwise, of any of the alumni of this College, that may be of interest to the Association, to be read at any future meeting, or to be placed upon the records of the Association, also to request all graduates to forward to him their names and address, to be entered upon the register, or, if any should at any time be in the city, to call and enter their uames themselves.

The Association was then invited to a reception to be held in the evening, after Commencement exercises, at the house of Dr. W. C. Hunt. The meeting then adjourned.

W. C. Hunt, Secretary.

